# Multilocular Radiolucent Pathology in the Body and Ramus of the Mandible: A Case Report

**DOI:** 10.7759/cureus.63722

**Published:** 2024-07-03

**Authors:** Mahek Batra, Swapnil Mohod, Prem A Sawarbandhe, Komal V Dadgal

**Affiliations:** 1 Department of Oral Medicine and Radiology, Sharad Pawar Dental College and Hospital, Datta Meghe Institute of Higher Education and Research, Wardha, IND

**Keywords:** multilocular radiolucent pathology, body of the mandible, odontogenic cyst, retromolar pad area, ramus of the mandible, odontogenic keratocyst

## Abstract

Odontogenic keratocyst (OKC), a type of epithelial developmental cyst, is frequently found in the jaw region. It has invasive characteristics such as satellite cysts, rapid progression, and tissue expansion. The OKC often favors the mandibular angle and ascending ramus. OKC symptoms include pain, swelling, displacement or malpositioning of adjacent teeth, and erosion or thinning of the limited or no bucco-lingual cortical expansion. There is radiographic evidence of a distinct, often scalloped, radiolucent lesion with a characteristic "soap bubble" or "honeycomb" appearance. This article reports a female patient, aged 40 years, with the main concern of unilateral pain and swelling of the mandibular left side and the provisional diagnosis of ameloblastoma. After histopathological examination, the final diagnosis of the patient was OKC. This article also includes previously published literature on OKC with differential diagnosis and relevant clinical and radiologic findings of the case.

## Introduction

Oral and maxillofacial tissues are impacted by a condition called odontogenic cysts. They originate from pathologic, developmental, or inflammatory factors related to the tooth-forming apparatus's odontogenic epithelium. Odontogenic keratocysts (OKCs), dentigerous cysts, periapical cysts, and residual cysts are the four categories of odontogenic cysts that develop most commonly [1]. Odontogenic cysts can be categorized as developing or inflammatory and are typically found during routine radiographic examinations. They appear as distinct-bordered, unilocular, or multilocular radiolucent lesions on radiography. However, they cannot be distinguished from one another. Furthermore, odontogenic cysts and aggressive odontogenic tumors may have comparable radiographic features [2].

The origin of OKC lies in the remnants of the dental lamina within the maxilla and mandible before the completion of odontogenesis. It might also come from the basal cells of the epithelium that covers it. In 1876, OKC was first recognized and documented. In 1956, Phillipsen categorized it further. Pindborg and Hansen proposed the histological criteria in 1962 that are required for the diagnosis of OKC. The World Health Organization (WHO) has suggested designating this lesion keratocystic odontogenic tumor (KCOT) instead of cystic neoplasm since this name more accurately describes the aggressive clinical behavior of the lesion, the high mitotic rate on histology, and the correlation with chromosomal and genetic abnormalities [3].

In 2005, it was later named OKC [3]. A thin, 5-8-cell-thick para-keratinized stratified squamous epithelium, held in place by a ridged layer of parakeratin, is one of its prominent histological characteristics. The basal cell layer displays a pattern of palisaded, homogeneous nuclei. One important aspect of OKCs is the production of satellite cysts, which are formed by the basal layer budging into the surrounding connective tissue. Usually devoid of inflammatory cell infiltration, the fibrous cyst wall is rather thin [4]. The para-keratinized and ortho-keratinized OKCs were found in comparable locations, more often in the mandible. The midline was where ortho-keratinized OKCs were more common than para-keratinized OKCs. That was the only discernible difference [5].

Pain, edema, and discharge are common complaints among patients with OKC. They sporadically suffer from lower lip paresthesia. Until the lesions grow big or have pathological fractures, some people are unaware of them. On radiographic evaluation, certain OKCs can be diagnosed without indication. Until the cysts develop significantly, patients are frequently remarkably unaffected by symptoms. The cyst has the same potential to cause tooth displacement as other intraosseous jaw lesions. Lund has reported the development of massive OKC involving the maxillary sinus, which results in proptosis of the eyes and destruction of the orbital floor [6]. The aggressive tendency of OKC resulted in the involvement of the surrounding soft tissues and the aperture of the cortical bone [7]. Patients may occasionally have symptoms including loose teeth, abnormal discharge, odd sensations, resulting in facial asymmetry, and discomfort. The most prevalent clinical symptom of OKC among them is edema. A facial malformation will arise if an OKC grows and the surrounding bone eventually grows. The surface bone consequently thins into a fragile bone plate and produces the so-called parchment-like, brittle sound [8].

The maxillary sinus and nasal cavity may potentially be invaded by the tumor cells in OKC. These cells have the potential to impair eyesight and perhaps seriously cause diplopia. The teeth may move, loosen, or tilt if OKC is close to them and compresses those teeth. Consequently, clinical tooth loss may coexist with OKC [8]. Comparable radiographic features, including a distinct multilocular or unilocular radiolucency associated with or independent of an unerupted tooth, may be seen in OKC and ameloblastoma instances. Comparatively speaking to ameloblastoma, OKC is less likely to induce root resorption and tends to develop along the interior part of the mandible, generating limited growth [9].

## Case presentation

A 40-year-old female reported to the Outpatient Department of Oral Medicine and Radiology, Sharad Pawar Dental College and Hospital, Wardha, with a chief complaint of pain in the lower left region of the jaw for the past six months. The pain was dull, aching, and intermittent, which gets aggravated when chewing as well as stimulated by hot and cold stimuli. The pain relieves itself on its own. The patient also experienced swelling in the left region of the jaw two months ago, which was initially smaller in size and gradually progressed to its current size. The patient did not give a history of hot and cold compression on the swelling. The patient visited a private dental clinic for pain and swelling, where the dentist took a small yellow color abnormal discharge sample from the left posterior region two months ago. After a few days, the swelling subsided, as told by the patient. The patient had a history of peptic ulcers 20 years ago and was on medication for them. The patient did not provide details about medications. The patient reported a prior extraction in the left-back region of the jaw one year ago at a private dental clinic, which was uneventful. The patient is not allergic to any drug known to him until now.

**Figure 1:**
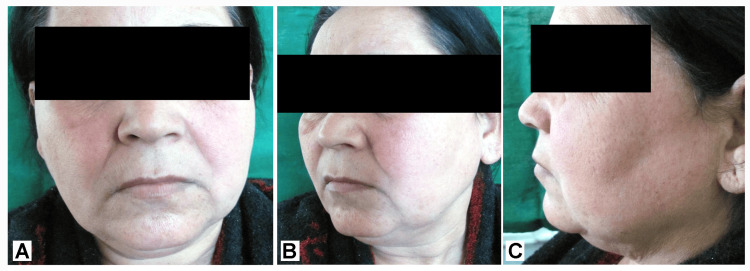
Extraoral examination showing bilateral symmetry of the face Image Credit: Swapnil Mohod

On extraoral examination, no gross asymmetry of the face was seen, as shown in Figure 1. Temporomandibular joint movements were bilaterally smooth and synchronous. No tenderness was present on palpating the temporomandibular joint and its associated muscles. A single, firm, mobile, and tender lymph node in the left submandibular area, measuring roughly 0.5×0.5 cm, was palpable.

**Figure 2:**
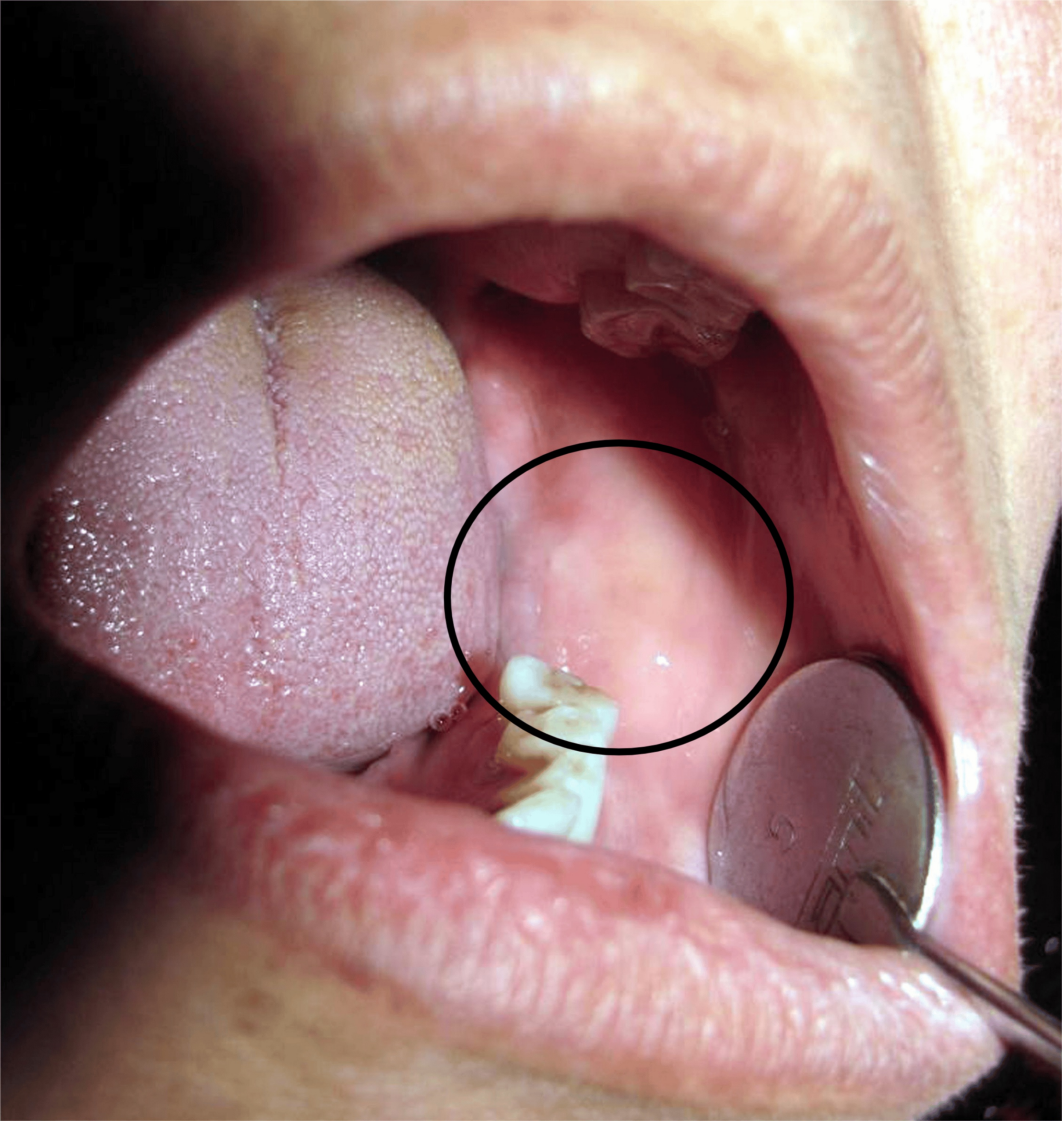
Intraoral examination showing diffuse swelling (36-38), which was obliterated on the lower gingival sulcus Image Credit: Swapnil Mohod

On intraoral examination, diffuse swelling was seen with 36-38, which was obliterated on the lower gingival sulcus in the 36, 37, and 38 regions of the jaw, extending anterior-posteriorly on the distal side of 35 to the retromolar region of the mandible and superior-inferiorly to the crest of the residual alveolar ridge to the depth of the vestibule. The size of the swelling was approximately 5×4 cm, with a roughly oval shape and diffused margins. The overlying mucous membrane was normal, as shown in Figure 2. On palpation, tenderness was present. There was no pus discharge on manipulation of the swelling. On clinical examination, a provisional diagnosis of "ameloblastoma involving the left side of the mandible" was given.

**Figure 3:**
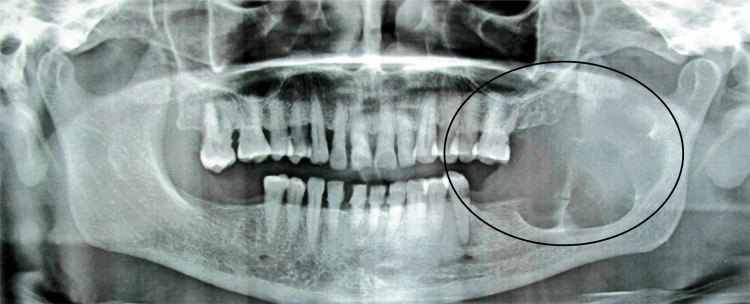
The orthopantomogram of the patient showing well-defined multilocular radiolucency on the left side of the mandible

The orthopantomogram of the patient in Figure 3 shows well-defined multilocular radiolucency on the left side of the mandible, extending anterior-posteriorly from the 36 to the left ramus of the mandible and superior-inferiorly from the superior aspect of the left ramus of the mandible to 5 mm above the lower border of the mandible of approximately 6×5 cm, roughly oval in shape, and well-defined and corticated margins. Internal structures were radiolucent. The radiographic diagnosis of the patient was OKC. The patient was advised to undergo a biopsy.

**Figure 4:**
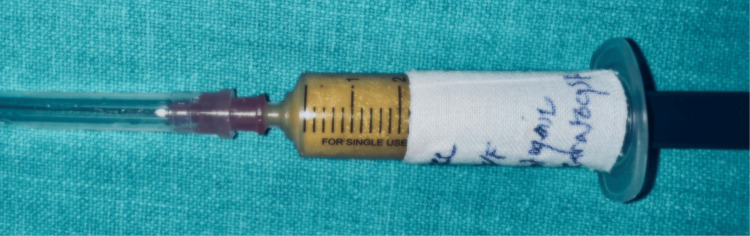
The aspiration of the swelling in the left region was done, and the fluid was thick yellow-colored with multiple tiny white granules

Prior to the procedure, informed consent was obtained from the patient after a detailed discussion regarding the surgery. The patient received comprehensive information about the potential recurrence of the lesion and the possibility of a pathological fracture. Under all aseptic conditions, the patient was prepared for the surgery. The inferior alveolar nerve block was given, and a full-thickness mucoperiosteal flap was reflected with no. 15 carbon steel surgical blade on the most prominent part of the left retromolar region, and the underlying bone was exposed. Cystic lining was curetted, and a small part of the bone was removed by C166 Edenta Lindemann Oral Surgery carbide bur under normal saline irrigation. Enucleation was done, and Carnoy's solution was used to chemically cauterize the bone cavity of the lesion. The aspirate from the cavity was sent for histopathological examination, as shown in Figure 4.

**Figure 5:**
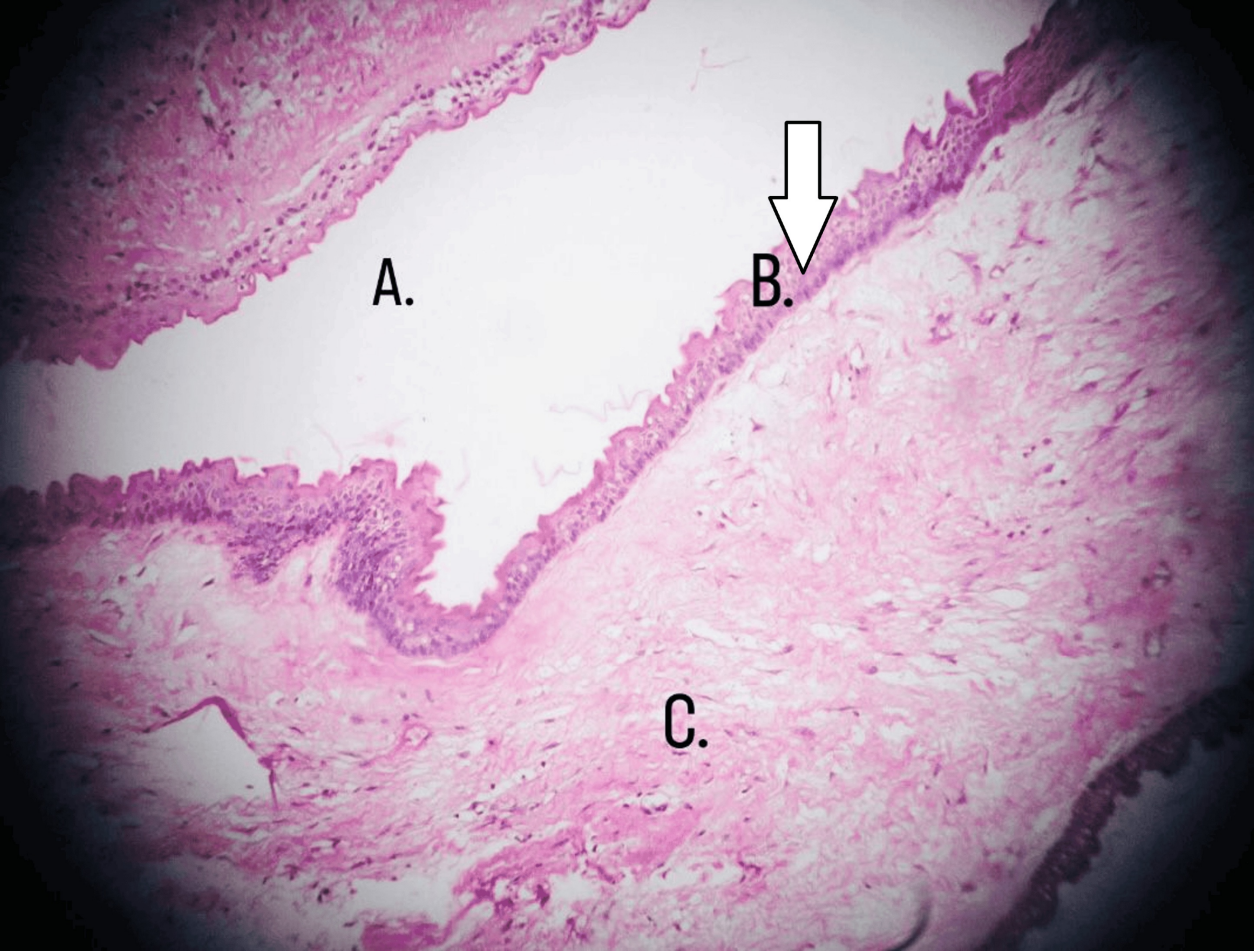
The histopathological report of the patient which is suggestive of odontogenic keratocyst: (A) cystic cavity/lumen, (B) thin para-keratinized stratified squamous epithelium, and (C) connective tissue

Repositioning of the flap was done, and the incision was closed with a 3.0 absorbable surgical vicryl suture (braided coated polyglactin). The patient was on antibiotics for five days. The antibiotics advised to the patient were amoxicillin 500 mg three times a day (TDS) and metronidazole 400 mg TDS for five days. The analgesic advised to the patient was diclofenac 100 mg twice daily for five days. The patient was also advised to take warm saline gargles and 0.2% chlorhexidine mouthwash three times daily for five days. The patient was instructed to consume a bland diet and periodic hydration, maintain good oral hygiene, and limit sugar and acidic foods. The patient was recalled after five days to monitor the healing of the bone and early recurrence. Microscopic examination of the lesion shows a cystic cavity lined by thin para-keratinized stratified squamous epithelium supported by a connective tissue wall. Surface corrugation of the keratinized layer was observed. Basal cells of the lining epithelium are columnar with polarized hyperchromatic nuclei resembling "tombstone." Histopathological features are characteristic of OKC, as shown in Figure 5.

**Figure 6:**
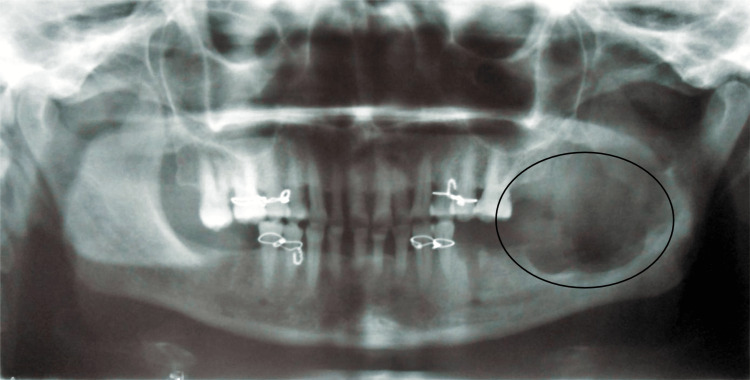
Post-operative radiograph showing surgical defect involving the left side of the mandible extending from 35 to the ramus region

A post-operative radiograph, as shown in Figure 6, shows a surgical defect involving the left side of the mandible, extending from 36 to the ramus region. Other radiographic findings were interdental wiring seen with 15-16, 24-25, 33-34, and 44-45 regions for the mobility of the teeth. Splinting of teeth is done to stabilize loosened teeth, promote healing of the supporting structures, restore proper function and occlusion while reducing pain, and prevent pathological fracture. Considering the close proximity of the lesion with caries involving periapical infection, tooth 35 was extracted.

**Figure 7:**
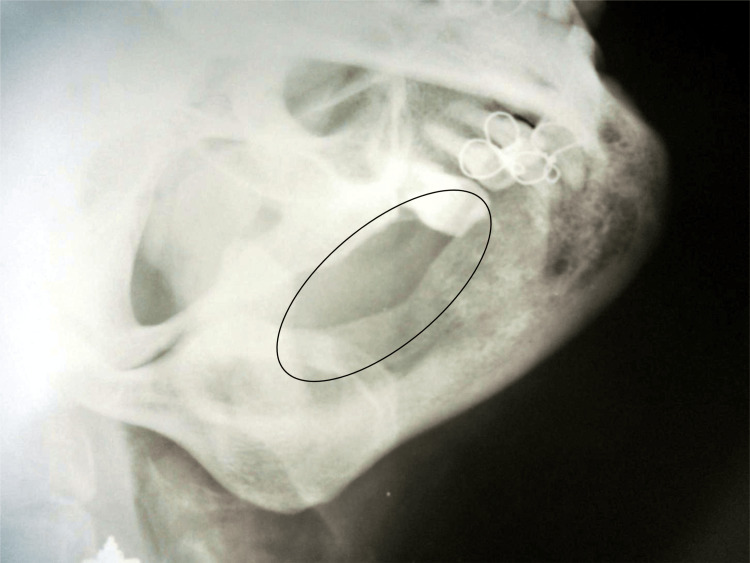
Follow-up left lateral oblique ramus-body projection radiograph after six months to inspect for recurrence of the lesion. There was no evidence of recurrence of the lesion

The patient was recalled for monthly follow-up for six months to inspect for recurrence of the lesion. There was no evidence of a recurrence of the lesion, as seen in Figure 7.

## Discussion

The recurrence rate of an OKC ranges from 12.3% to 58.3%. Several factors could account for this wide variability, including differences in case numbers and observation durations, the incorporation of lesions featuring ortho- or para-keratinized epithelium, and the inclusion of lesions associated with basal cell nevus syndrome. The existence of satellite cysts, epithelial remains, and inadequate excision of the original lesion with a thin epithelial lining are assumed to be the causes of the high recurrence rate of OKC, while the actual cause is unknown [10]. OKC was shown to occur across a wide range of age groups. There was a 50% male predisposition in the ratio of males to females with OKC, which was 1.42. Pain, edema, and both were the most often seen clinical symptoms. It was discovered that the most frequent OKC location was the mandible, revealing a noteworthy variation in the recurrence rates among the age groups. Compared to patients in the other age categories, patients in their fifth decade of life had noticeably greater rates of OKC recurrence [11].

In oral and maxillofacial surgery, there is considerable debate over the management of OKC. The management of OKC is influenced by the patient's age, accessibility, size of the cyst, surrounding connective tissue, proximity to the mandibular nerve, cortical bone penetration, and cyst recurrence. Numerous therapeutic modalities were employed, including decompression, electrocautery, and enucleation with adjuvant treatments (Carnoy's solution, cryotherapy, or peripheral osteotomy), although many of these techniques could potentially harm nearby tissues. To lower the probability of recurrence, a few surgeons advise peripheral osteotomy, an aggressive type of adjuvant therapy in which any cystic remains of the bony cavity are stained with methylene blue using a bone bur. Liquid nitrogen cryotherapy causes the epithelium to necrotize. Many prefer to chemically cauterize the bone cavity (Carnoy's solution is composed of 1 g of ferric chloride dissolved in 6 mL absolute alcohol and 1 mL glacial acetic acid) after cystic excision. To remove the cyst more easily and with a reduced chance of recurrence, Carnoy's solution has also been injected intraluminally to liberate it from the bone wall. This is a good first line of therapy with a low chance of recurrence. A modified marsupialization procedure called decompression results in a considerable reduction in the size of the cyst and thickening of the cystic lining, which is similar to the oral mucosa and facilitates simpler enucleation. IL-1α, which controls OKC epithelial cell growth, is reduced by this technique. Decompression followed by enucleation was found to dramatically reduce the recurrent rate of OKC when compared to enucleation alone. The non-surgical treatment of OKC has drawn the attention of certain researchers recently. A hedgehog pathway inhibitor (vismodegib) is used orally (150 mg/day for 18 months) to cause cystic shrinking as a more targeted form of treatment for individuals with numerous, recurring, or expansive OKC [12].

OKCs have a distinctive para-keratinized stratified epithelial lining that is 6-11 cells thick under the microscope. Usually, the lining epithelium has a corrugated surface. The tall columnar cells that line the basal layer have a nuclear palisading pattern. There is flat contact between the epithelium and the fibrous wall, and there is also evidence of partial epithelium separation. The fibrous wall has a persistent inflammatory cell infiltration [1].

## Conclusions

OKCs are aggressive jaw cysts that develop from dental lamina remains. They have a high recurrence rate. They necessitate proper diagnosis by imaging and histology, and their treatment frequently entails surgical intervention with long-term monitoring. Advances in molecular biology give hope for tailored treatments. OKC therapy requires a cautious approach to reduce recurrence and related problems. Patients with OKC should maintain regular follow-up sessions to monitor recurrence. Post-treatment care entails practicing excellent oral hygiene, quickly reporting any symptoms or changes, and adhering to the dental or surgical care team's recommendations. Long-term attention is required to detect and handle any recurrences.
